# Dormant glioblastoma cells acquire stem cell characteristics and are differentially affected by Temozolomide and AT101 treatment

**DOI:** 10.18632/oncotarget.22514

**Published:** 2017-11-18

**Authors:** Vivian Adamski, Annika Hempelmann, Charlotte Flüh, Ralph Lucius, Michael Synowitz, Kirsten Hattermann, Janka Held-Feindt

**Affiliations:** ^1^ Department of Neurosurgery, University Medical Center Schleswig-Holstein UKSH, Campus Kiel, 24105 Kiel, Germany; ^2^ Department of Anatomy, University of Kiel, 24118 Kiel, Germany

**Keywords:** cellular dormancy, stemness, cellular plasticity, chemoresistance, R-(-)-gossypol

## Abstract

Cellular dormancy is defined as a state in which cells enter quiescence driven by intrinsic or extrinsic factors, and striking parallels exist between the concept of cellular dormancy in malignancies and the cancer stem cell theory. We showed now that the proven dormancy markers insulin-like growth factor-binding protein 5, ephrin receptor A5 and histone cluster 1 H2B family member K were expressed in human glioblastomas *in situ*, were located in single tumor cells, and could be co-stained with each other and with the stem cell markers krüppel-like factor 4, octamer binding transcription factor 4 and sex determining region Y-box 2. Human non-stem glioblastoma cell lines and primary cultures were characterized by expression of individual, cell-type specific dormancy- and stemness-associated markers, which were (up)regulated and could be co-stained in a cell-type specific manner upon Temozolomide-induced dormancy *in vitro*. The induction patterns of dormancy- and stemness-associated markers were reflected by cell-type specific responses to Temozolomide-induced and combined Temozolomide/AT101-mediated cytotoxicity in different glioblastoma cell lines and primary cultures *in vitro*, and accompanied by higher self-renewal capacity and lower TMZ-sensitivity of Temozolomide-pretreated cells. We postulate that a better understanding of the dormant state of tumor cells is essential to further improve efficiency of treatment.

## INTRODUCTION

Gliomas represent the majority of primary brain tumors in adults, and the most malignant form, *glioblastoma multiforme* (GBM), accounts for more than 15% of all intracranial tumors [1]. The typical hallmarks of cancers [2] are also present in GBMs and favor tumor development and progression.

As a novel, additional hallmark, cancer dormancy has recently been postulated [3]. Cancer dormancy is a widely described phenomenon of malignant tumors addressing a protected state which may occur at different stages of tumor progression or after an apparently successful therapeutic intervention [4]. In addition to well-known angiogenic and immunogenic dormancy processes, there also exists a dormant, resting state on the cellular level within the tumor [5]. This cellular dormancy is defined as a state in which either solitary or small groups of cells enter quiescence (reversible growth arrest) driven by intrinsic or extrinsic factors [6]. Dormant tumor cells are highly prevalent in the general population [4], and dormant tumor cells remaining after primary tumor removal or treatment are commonly refractory to chemotherapy [4, 6].

Interestingly, striking parallels exist between the concept of tumor dormancy and the cancer stem cell theory [7]. Moreover, recent data indicate that stem cell properties are not fixed to particular cells but can be gained and lost in dependence on the microenvironment [8].

Recently, the existence of tumor dormancy has also been proven in gliomas as a subfraction of dormant tumor cells was detected in a mouse GBM model [9]. Additionally, some tumor cell lines including GBM lines failed to induce tumors *in vivo* for a long period [10]. Furthermore, expression analysis between dormant and fast growing phenotypes of GBM cells revealed that a specific gene set is upregulated in dormant GBMs, including e.g. ephrin type-A receptor 5 (EphA5), thrombospondin, angiomotin, insulin-like growth factor-binding protein 5 (IGFBP5), and histone cluster 1 H2B family member K (H2BK) [11, 12].

A possible connection between the tumor dormancy concept and the cancer stem cell theory in GBMs has not been proven by now. However, a first study shows the induction of stem cell markers [e.g. octamer binding transcription factor 4 (OCT4), sex determining region Y-box 2 (SOX2), nestin, CD133] in a subfraction of non-proliferating cells in a mouse GBM model [9].

Now, we investigated the phenotypic switching to cellular dormancy and a putative link to stem-like characteristics in GBM *in situ* and *in vitro*, and observed individual responses of different cells to combined treatment strategies employing Temozolomide (TMZ) and AT101, an inhibitor of antiapoptotic Bcl-2 family proteins.

## RESULTS

### Co-expression of dormancy- and stemness-associated genes in GBMs in situ

First, we investigated the occurrence of dormant cells in solid human glioma samples using proven dormancy markers [11], and characterized them comprehensively according to their co-expression with stem cell markers *in situ*.

**Figure 1:**
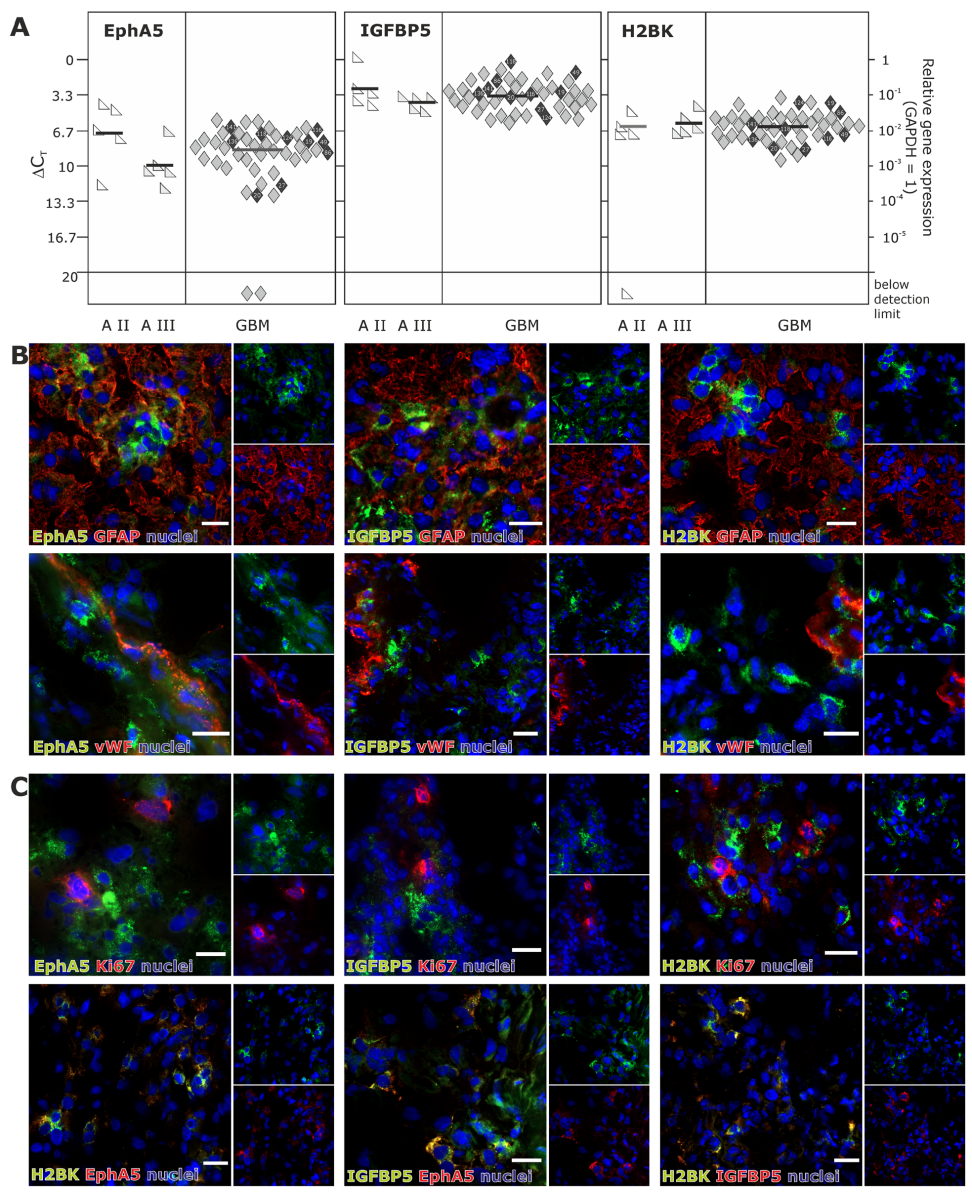
Expression of EphA5, IGFBP5 and H2BK in solid human glioblastoma (GBM) samples **(A)** Solid human glioma samples of different tumor entities were analysed by qRT-PCR regarding the expression of EphA5, IGFBP5 and H2BK (A = astrocytoma, GBM = glioblastoma). Lines represent the mean gene expression for each gene and tumor entity (ΔCT 3.3 = 10-fold expression difference; black-marked rhombs correspond to cultured GBM cells in Figure 3A). (**B** and **C**) Solid human glioblastoma sections were stained by double-immunohistochemistry regarding the expression of IGFBP5, EphA5 and H2BK to identify dormant cells. Sections were also stained by double-immunohistochemistry with combinations of (**B**) GFAP, vWF or (**C**) Ki67 and with IGFBP5, EphA5, H2BK themselves (white bars indicate 20 μm).

**Figure 2:**
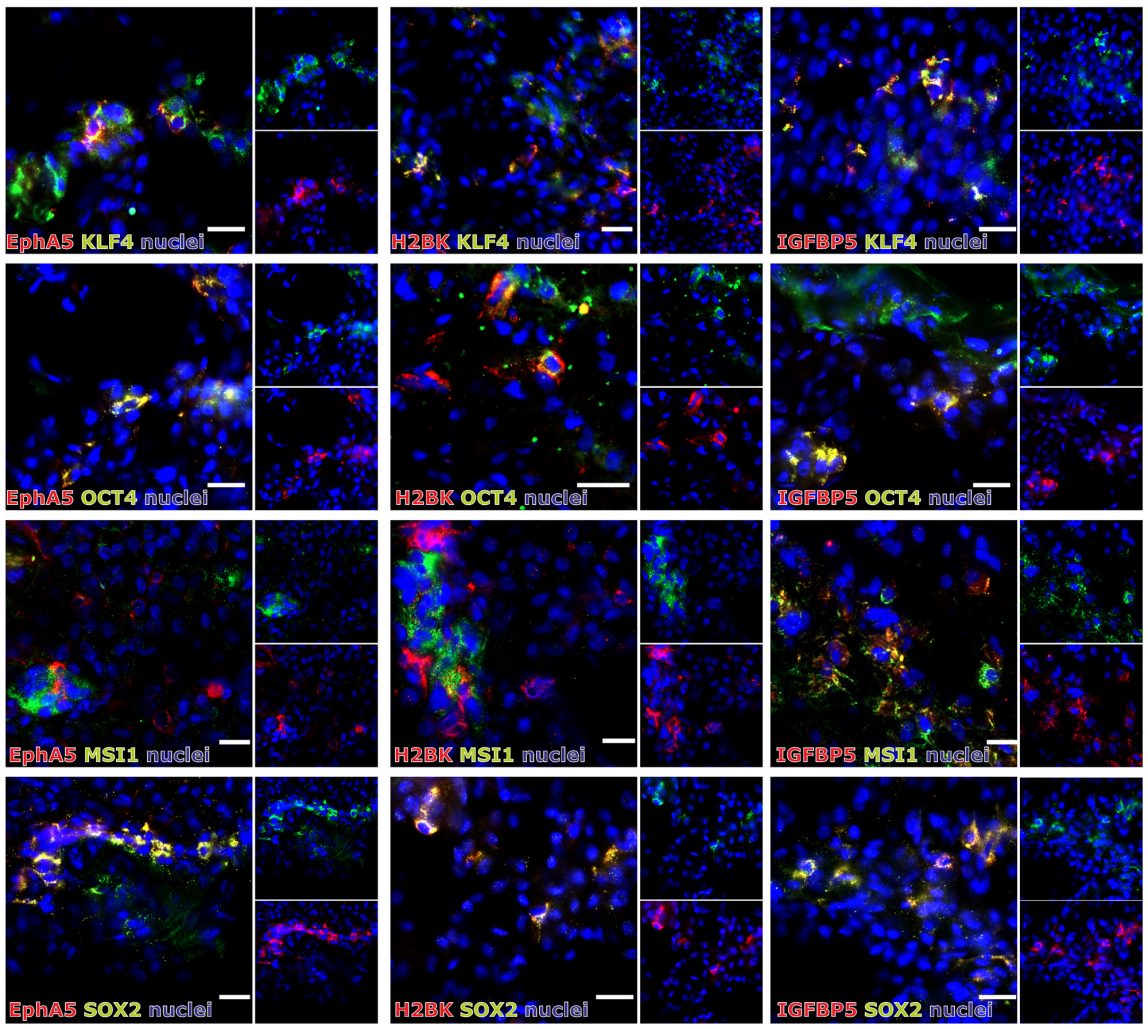
(Co)-Expression of EphA5, IGFBP5 and H2BK with the stem cell markers KLF4, OCT4, MSI1 or SOX2 in solid human GBM samples Solid human GBM sections were stained by double-immunohistochemistry for IGFBP5, EphA5 and H2BK in combinations with KLF4, OCT4, MSI1 or SOX2 (white bars indicate 20 μm).

In solid human astrocytomas (A) WHO grade II and III as well as in GBM samples EphA5, IGFBP5 and H2BK were expressed (with single exceptions) on well detectable levels without any widespread differences between individual tumor entities [Figure 1A; average ΔCT values for GBM samples: 8.47 (EphA5), 3.40 (IGFBP5), 6.29 (H2BK)]. When visualizing EphA5, IGFBP5 and H2BK protein expression by fluorescence staining in solid human GBM samples, all molecules were stained in solitary cells or small cell groups within glial fibrillary acidic protein (GFAP, glial marker) positive tumor regions and (near) beside von Willebrand factor (vWF, endothelial marker) positively stained tumor vessels (Figure 1B). However, by double-immunoflourescence staining with CD11b (CD11b is also known as Mac-1 α or integrin αM chain; expressed on myeloid cells, NK cells, some activated lymphocytes as well as on microglia in the brain), we could exclude solely expression on immune cell types (not shown). Since dormancy should indicate a resting cell state, EphA5, IGFBP5 and H2BK were not co-expressed with the proliferation marker Ki67, but were found to be co-stained with each other to a high extent (Figure 1C). In addition, we investigated co-staining of dormancy markers EphA5, IGFBP5 and H2BK with selected neural and embryonic stem cell markers that we and others have previously shown to be relevant in gliomas [13]. As shown in Figure 2, dormancy-associated genes were especially co-stained with the stem cell markers krüppel-like factor 4 (KLF4), octamer binding transcription factor 4 (OCT4) and sex determining region Y-box 2 (SOX2) in GBM samples. A co-staining with Musashi-1 (MSI1) was also observed but not as prominent as detected for the other investigated stem cell markers. Nevertheless, several dormancy and stem cell marker single positive or negative cells, respectively, could be recognized within the sections.

### Induction of cellular dormancy using TMZ in non-stem GBM cells *in vitro*

To further evaluate a possible connection between GBM cellular dormancy and stem cell characteristics, in a next step we transferred the *in situ* results to *in vitro* cultured GBM cells. Since we wanted to focus especially on chemotherapy-induced cellular dormancy in this context, in a first step we established an *in vitro* model of dormant GBM cells which was useful for our further investigations.

**Figure 3:**
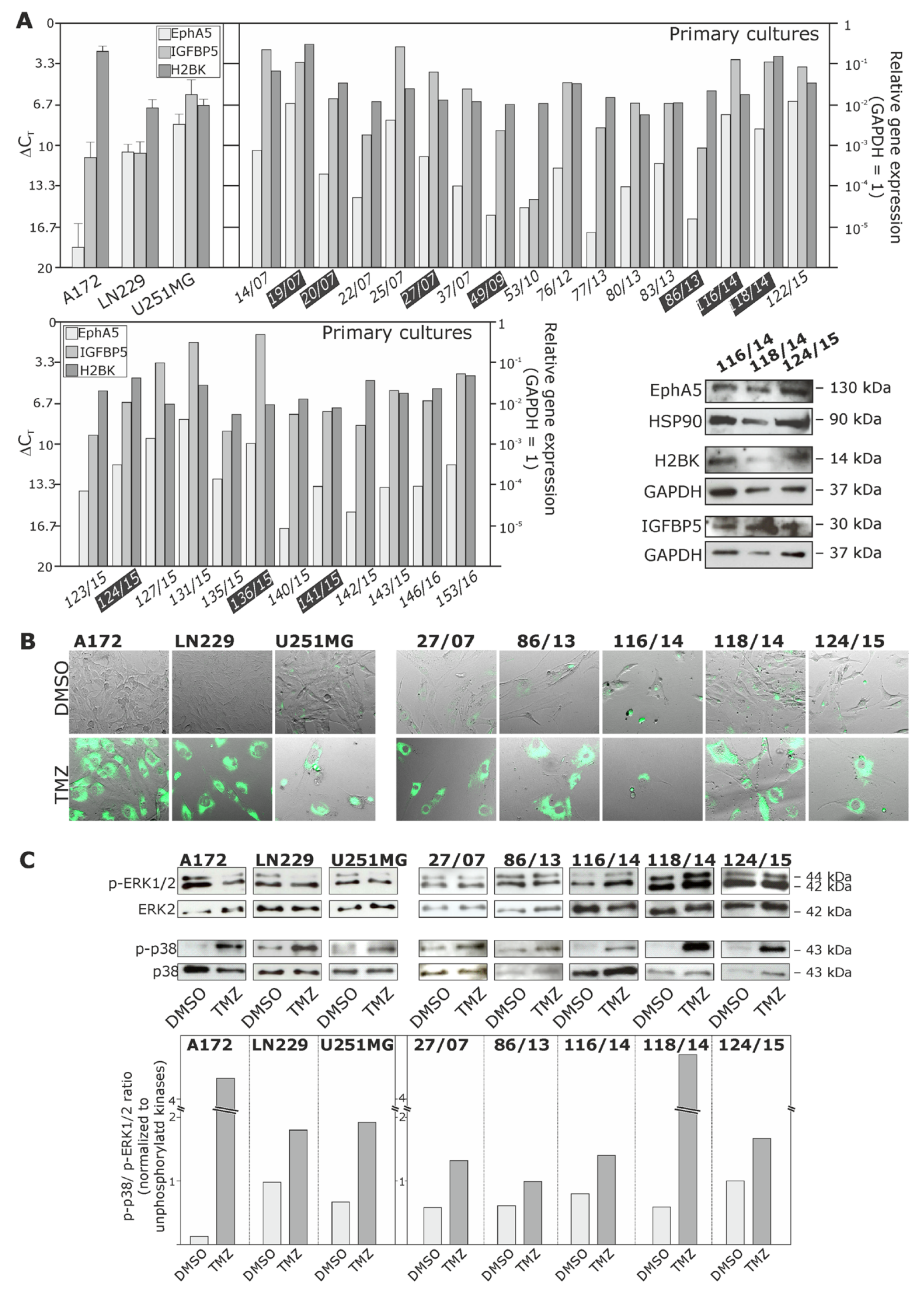
Expression of EphA5, IGFBP5 and H2BK in cultured human non-stem GBM cell lines and primary cultures, and analysis of a Temozolomide (TMZ)-induced cellular dormant state in different GBM cultures **(A)** Cultured human glioma cell lines and primary cultures were analysed by qRT-PCR and Western Blot regarding the mRNA and protein expression of EphA5, IGFBP5 and H2BK (ΔCT 3.3 = 10-fold expression difference; black highlighted primary cultures correspond to solid GBM samples in Figure 1A). **(B** and **C)** GBM cells were stimulated with 500 μM TMZ or 0.2% DMSO (control) for 10 days, and the dormant state was analysed by monitoring dye retention at day 10 using combined transmitted-light and fluorescence microscopy **(B)**, and determination of phospho-p38 / phospho-p42/44 ratios by Western Blot and subsequent densitometric analysis comparing DMSO and TMZ treated samples **(C)**.

Initially, we determined the basal mRNA and protein expression of EphA5, IGFBP5 and H2BK in human non-stem glioma cell lines (A172, LN229 and U251MG) and several GBM primary cultures (basal expression of stem cell markers has been described by our group before [13]). Although these dormancy-associated molecules were found in individual and different amounts, GBM cultures were characterized by a clear mRNA (quantitative PCR) and protein (Western Blot, immunocytochemistry) expression of EphA5, IGFBP5 and H2BK (Figure 3A, black highlighted primary cultures numbers correspond to solid GBM samples depicted in Figure 1A; Figure 7A and 7B). Next, we stimulated known TMZ-sensitive GBM non-stem cell lines (A172, LN229 and U251MG) [14, 15] and several primary cultures (27/07, 86/13, 116/14, 118/14, 124/15) with TMZ for up to 10-12 days. TMZ itself is a common GBM chemotherapeutic which is known to induce G2/M cell cycle-arrest [16]. Subsequently, we verified the induction of a dormant state by DiO retention labeling and analysing phospho-p38 / phospho-p42/44 ratios. Since the fluorescence intensity in cycling cells decreases by half due to cell division, fluorescence label-retaining assays can effectively discriminate dormant or slow-cycling cells from fast-cycling cells [17]. In addition, an adjustment of phospho-p38 / phospho-p42/44 ratios to higher phospho-p38 extents is well known to be associated with a dormant state [18].

As obvious, during a permanent 10-12 days TMZ stimulation time cultured glioma cell lines and primary cells died to individual extents. However, some GBM cells were able to overcome TMZ treatment and were mainly characterized by a vast morphology with large nuclei. Surviving cells were characterized by DiO retention and, to individual extents, by increased phospho-p38 / phospho-p42/44 ratios (exemplified in Figures 3B and 3C; 10 day TMZ stimulations in comparison to DMSO controls). In addition, TMZ-pretreated (10-12 days) GBM cells showed lower TMZ-response in sequel when stimulated with an additional TMZ-treatment (for another 10 days). Here, TMZ-sensitivity was measured by comparison of TMZ-pretreated and native (untreated) GBM cells using cytotoxicity assays. In detail, normalized to whole cell counts, 50.2±4.4% dead cells were found in native GBM cells whereas in TMZ-pretreated cultures only 41.3±7.2% dead cells were detected after an additional TMZ treatment for 6 days, and further 31.7±2.3% native and 17.7±6.5% pretreated dead cells were detected after an additional TMZ treatment for 10 days (n=3; not shown).

### Influence of TMZ on dormancy and stemness characteristics of GBM cells *in vitro*

**Figure 4:**
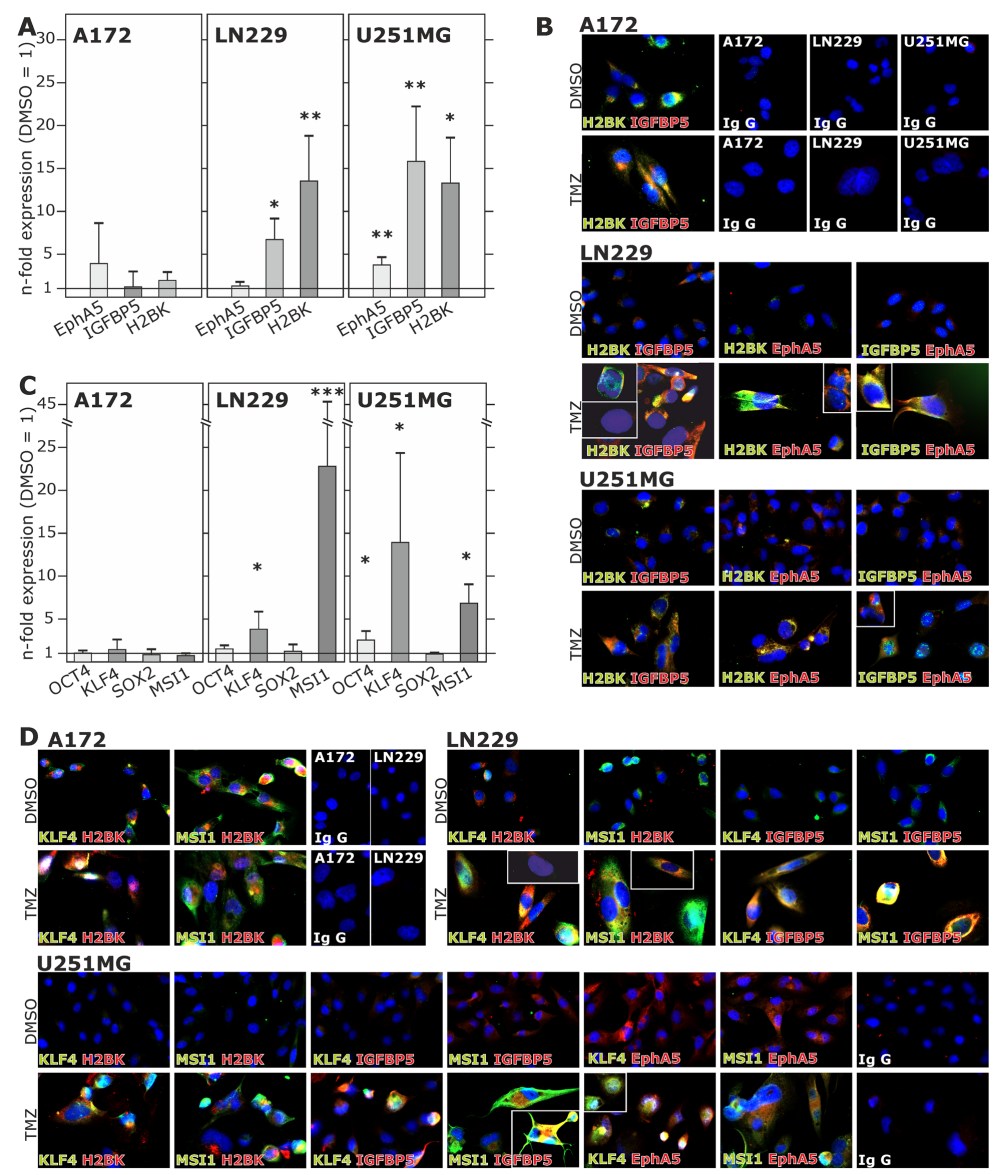
Induction and (co)-expression of dormancy- and stemness-associated genes during TMZ treatment in glioma cell lines (**A** and **C**) Non-stem GBM cell lines were stimulated with 500 μM TMZ or 0.2% DMSO (control) for 10 days and EphA5, IGFBP5 and H2BK (A) and OCT4, KLF4, MSI1 or SOX2 (C) expression was analysed in TMZ-stimulated compared to control samples using qRT-PCR (^*^p<0.05, ^**^p<0.01, ^***^p<0.001). **(B** and **D)** Stimulated GBM cell lines were stained by immunocytochemistry to analyse the (co)-expression of IGFBP5, EphA5, H2BK themselves **(B)** and together with KLF4 and MSI1 **(D)**. IgG antibodies served as secondary antibody controls.

**Figure 5:**
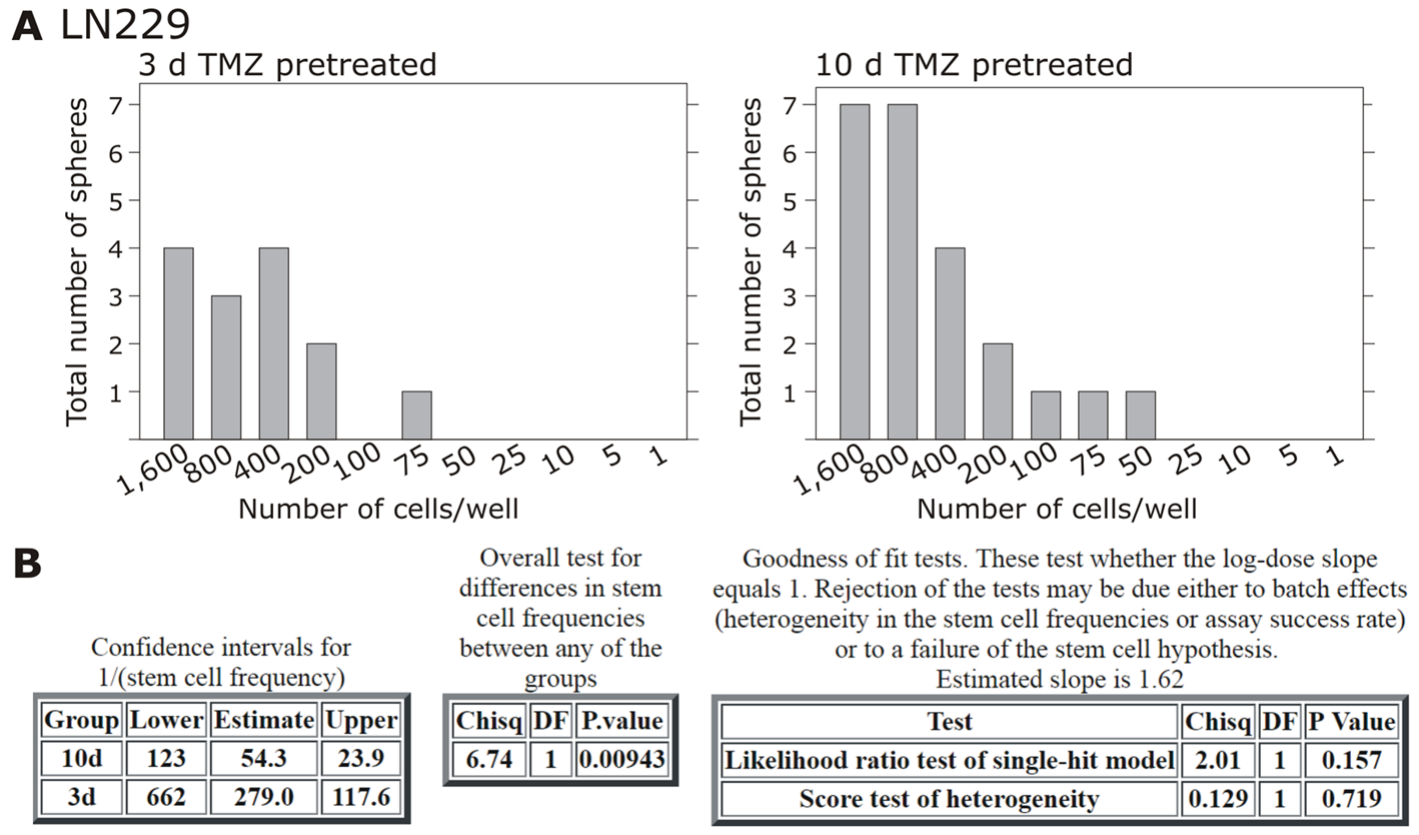
Extreme limiting dilution analysis (ELDA) and self-renewal capacity Non-stem LN229 cells were stimulated with 500 μM TMZ for 10-12 or 3 days, and according to acquired induction of stem cell markers self-renewal capacity was determined under stem cell culture conditions with ELDA (n=2). Briefly, cells were plated in decreasing numbers from 1,600 cells / well to 1 cell / well. Cultures were maintained until day 10, when the number of spheres per well **(A)** and wells containing spheres for each cell plating density (number of positive cultures) were recorded and plotted using online ELDA program25 **(B)**; http://bioinf.wehi.edu.au/software/elda.

**Figure 6:**
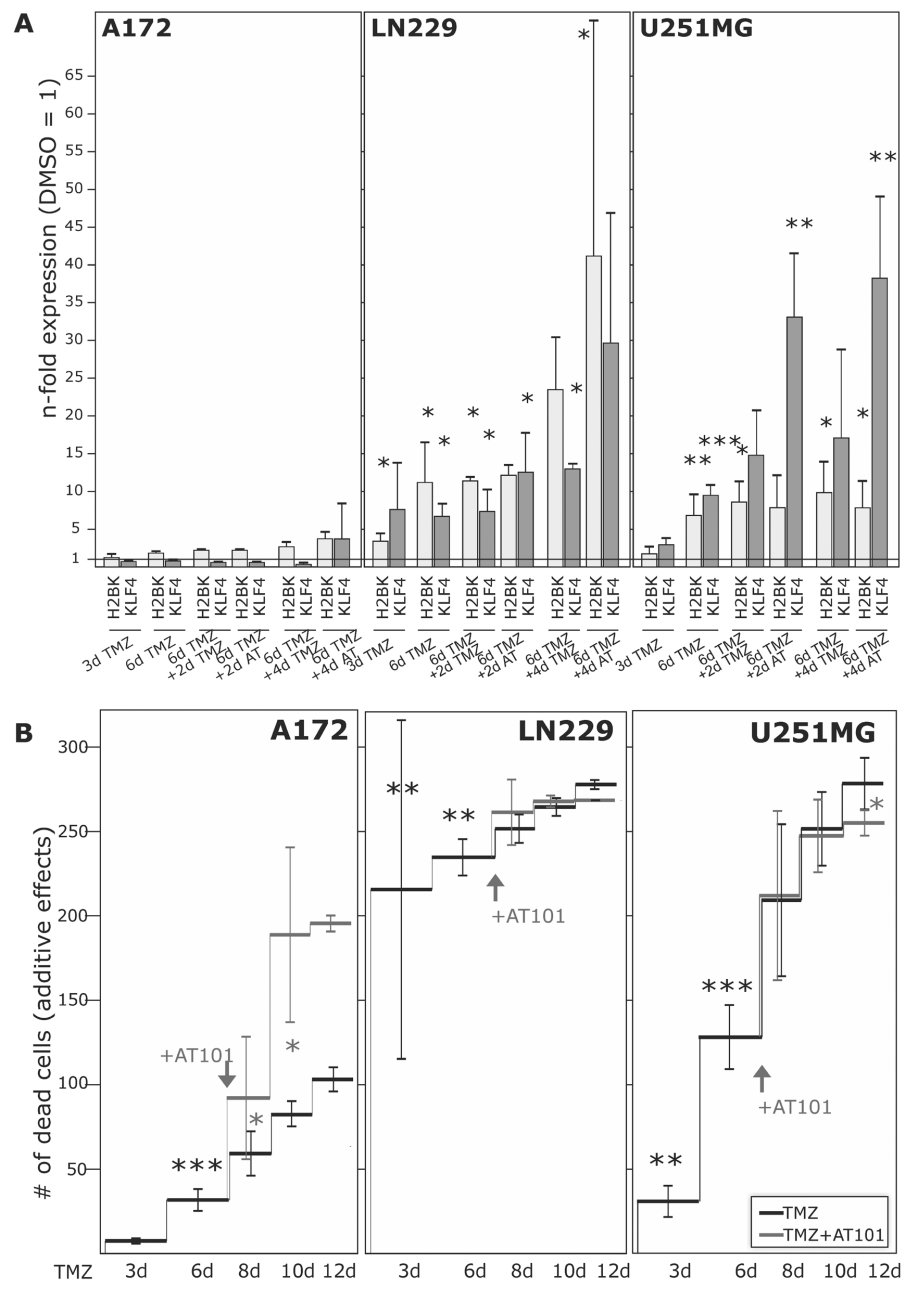
Induction of dormancy- and stemness-associated genes during TMZ treatment in glioma cell lines, and determination of TMZ-induced and combined TMZ / AT101-induced cytotoxicity GBM cell lines were stimulated with 500 μM TMZ, 5 μM AT101 or 0.2% DMSO (control) for up to 10-12 days. 5 μM AT101 was added to TMZ stimulated cells at day 6, and stimulation was performed for additional 4-6 days. **(A)** Expression of H2BK and KLF4 was checked at days 3-6-8-10 using qRT-PCR, and **(B)** numbers of dead cells were determined at days 3-6-8-10-12 and documented as n-fold cytotoxic effects (^*^p<0.05, ^**^p<0.01, ^***^p<0.001).

**Figure 7:**
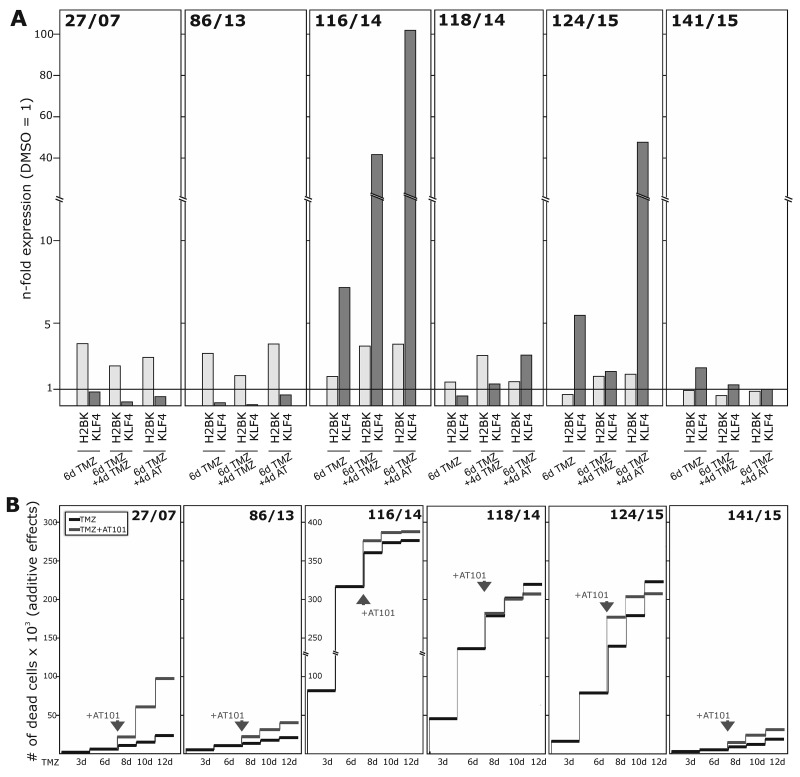
Induction of dormancy- and stemness-associated genes during TMZ treatment in GBM primary cultures, and determination of TMZ-induced and combined TMZ / AT101-induced cytotoxicity Primary cultures were stimulated with 500 μM TMZ, 5 μM AT101 or 0.2% DMSO (control) for up to 10-12 days. 5 μM AT101 was added to TMZ stimulated cells at day 6, and stimulation was performed for additional 4-6 days. **(A)** Expression of H2BK and KLF4 was checked at days 6 and 10 using qRT-PCR, and **(B)** numbers of dead cells were determined at days 3-6-8-10-12 and documented as n-fold cytotoxic effects.

Alongside with these functional effects of cellular dormancy, GBM cell lines and primary cultures showed an induction of dormancy-associated genes [Figure 4A/4B and Figure 6A for glioma cell lines, Figure 7A for primary cultures; 10 days (Figure 4A/4B) and 3-6-8-10 days (Figure 6A, 7A) time elapsed stimulations]. In these investigations, dormancy-associated gene expression was affected by TMZ-treatment only to a moderate degree in some GBM cells (A172, 27/07, 86/13 and 141/15), while other ones (LN229, U251MG, 116/14, 124/15) responded to higher extents, and expression of especially IGFBP5 and H2BK were induced considerably. Interestingly, expression analysis of some of the selected stemness-associated genes (clear induction of KLF4 and MSI1, slight/partly induction of OCT4, but no induction of SOX2) reflected the observed induction of dormancy-associated ones in a comparable manner (compare Figure 4C/4D and Figure 6A for glioma cell lines, Figure 7A for primary cultures). Here, especially expression of KLF4 and MSI1 were clearly induced upon TMZ treatment, and we chose the marker KLF4 to show that the extend of induction depended on the stimulation time (Figure 6A: LN229, U251MG; Figure 7A: 116/14, 118/14, 124/15). Beyond, as exemplified for LN229, long-term TMZ-pretreated cells exhibited higher self-renewal capacity as determined by comparison of sphere formation under stem cell culture conditions between 10 days *versus* 3 days TMZ-pretreated GBM cells (clear *versus* slight induction of stemness associated genes, compare 6A) in an extreme limiting dilution analysis (ELDA; Figure 5A/5B).

When investigating co-expression of different dormancy-associated genes by double-fluorescence staining, dormancy-associated markers were mostly co-stained in different cultures (Figure 4B). Due to (relatively) high induction of dormancy-associated genes in e.g. LN229 and U251MG, co-staining of these markers were particularly impressive in these two cell lines. Beyond, dormancy-associated markers were clearly co-stained with KLF4 and MSI1 in different GBM cells, respectively, and TMZ-mediated induction of both dormancy- and stemness-associated markers was well visible in identical cells (Figure 4D). However and in accordance with Figure 4A-4C, these results were particularly impressive for those dormancy and stemness genes and GBM cells, respectively, which responded most intensely to TMZ (Figure 4D). Nevertheless, single positive or complete negative cells existed in all investigated combinations, and staining intensities and differences between stimulated and unstimulated GBM cells varied due to different expression levels of dormancy- and stemness-associated genes.

### Effects of single and combined treatment strategies on dormant GBM cells *in vitro*

To analyse whether the differences in TMZ-mediated induction of dormancy- and stemness-associated genes were reflected by variable chemotherapeutic responses of investigated GBM cell lines and primary cultures, we determined the amounts of dying cells by cytotoxicity assays.

Firstly, we measured the influence of TMZ alone during a 10-12 day stimulation time period (Figure 6B and Figure 7B). Irrespective of a TMZ-sensitivity in general, the amounts of dying and surviving cells varied between individual cell lines (A172, LN229, U251MG) and primary cultures (27/07, 86/13, 116/14, 118/14, 124/15, 141/15). While LN229, U251MG, 116/14, 118/14 and 124/15 responded within the first days of TMZ treatment with high extents of dying cells, A172, 27/07, 86/13 and 141/15 reacted to (relatively) lower extents (exemplified in Figure 6B and Figure 7B – dark lines). Interestingly, in contrast to these later mentioned “bad responders”, the group of “good responders” included GBM cultures which were characterized by considerable induction of dormancy- and stemness-associated genes (exemplified by H2BK and KLF4 in Figures 6A and 7A) in a time-dependent manner in the surviving cells (Figure 6A: LN229, U251MG; Figure 7A: 116/14, 118/14, 124/15).

In a next step, we wanted to evaluate effects of a combined TMZ and AT101 treatment strategy on TMZ-induced dormant GBM cells with stem-cell characteristics. AT101, the R-(-)-enantiomer of Gossypol, is an inhibitor of antiapoptotic Bcl-2 family proteins, and might probably affect glioma cells that were in a dormant state due to TMZ treatment. To investigate this potential cytotoxic effect of AT101, GBM cells were stimulated for 6 days with TMZ alone, and afterwards for another 6 days with TMZ and AT101 together (treatment schedule in Supplementary Figure 2). Combined TMZ and AT101 treatment usually yielded high cytotoxic effects in GBM cells which responded to small extents to TMZ treatment (e.g. Figure 6B: A172; Figure 7B: 27/07), whereas in GBMs cells responding well to TMZ treatment (e.g. Figure 6B: LN229; Figure 7B: 116/14), additional AT101 treatment mostly induced only slight synergistic cytotoxic effects. In detail, combinational index values at FA of 0.5 (growth-inhibitory effect of each drug) of cytotoxic effects were calculated using the CompuSyn software yielding synergistic effects for A172 (0.6227) and U251MG cells (0.6680), as well as a slight synergism of drugs for LN229 (0.8815). Effects of AT101 alone, however, were individual in our investigated GBM cell lines and primary cultures, and singular AT101 treatment did not yield any clearly detectable morphological changes (Supplementary Figure 1 and Supplementary Figure 3; due to different cell amounts in TMZ / AT101 *versus* AT101 stimulations, scales indicating the n-fold cytotoxic effects are not directly comparable between respective assays). Interestingly, the induction of KLF4 in LN229, U251MG, 116/14, 118/14 and 124/15 persisted also during combined TMZ and AT101 time-course of stimulation (Figure 6A, Figure 7A).

## DISCUSSION

Cancer dormancy is defined as a stage in which tumors remain occult and asymptomatic for a prolonged period of time. Clinical data and experimental models have led to the development of two concepts – the cellular dormancy and whole tumor dormancy [4, 5, 12, 19]. Cellular dormancy is defined as a state in which either solitary or small groups of cells enter a reversible growth arrest driven by e.g. therapeutic intervention [5]. Some research groups were able to show that cancer dormancy also plays a prominent role in GBM progression [9–12, 20].

Interestingly, there are striking parallels existing between the cellular dormancy of malignancies and the cancer stem cell theory [7]. Both predict that a subset of tumor cells is responsible for tumor initiation, bears the ability to survive therapy, and may persist in a dormant state for longer time periods to cause delayed cancer recurrence and progression. Confirming the connection of these two theories, we showed now that the GBM-relevant known dormancy markers IGFBP5, EphA5 and H2BK were expressed in GBMs *in situ* and *in vitro* and could be co-stained with each other and with the stem cell markers KLF4, OCT4 and SOX2. In addition, dormancy and some stemness-associated markers were upregulated and could be co-stained in a cell-type specific manner upon TMZ-induced dormancy in non-stem cell cultures of primary and commercial human GBM cell lines. Here, occurrence and extend of regulation depended on the investigated cell lines and markers: later identified “good responders” seemed to react to greater extent, and predominantly KLF4 and MSI1 were induced alongside with dormancy-associated markers hinting to a special need for cellular plasticity to gain a dormant phenotype. In accordance with this, long-term TMZ-pretreated GBM cells were characterized by higher self-renewal capacity. Thus, a connection between the two theories seems probable, and a therapy-driven plasticity of non-stem glioma cells towards a more stem-like phenotype becomes obvious. Interestingly, evidence is emerging that non-stem cells can be transferred into a transient, drug-tolerant, stem cell-like state by chemotherapy [21, 22]. Complementary, hypoxia stimulates the self-renewal capability of a non-stem population in GBM and promotes a more stem-like phenotype with upregulation of different stem cell factors [23]. Furthermore, TMZ exposure to glioma xenograft lines consistently increased stem cell frequency over time [24]. Lineage tracing analysis revealed cellular plasticity within glioma cells, allowing them to reprogram from a differentiated to an undifferentiated stem-like state which is driven by TMZ-induced hypoxia inducible factors (HIFs). Similarly, TMZ therapy significantly increased the rate of single-cell conversions, and therapy-induced HIF1a and HIF2a seem to play key roles in allowing non-stem glioma cells to acquire stem-like traits [25].

To analyse how the cell death of GBM cell lines and primary cultures with TMZ-induced dormant and stem cell properties can be influenced by therapeutic interventions, we performed a combined chemotherapeutic strategy. We chose a sequential, combined TMZ/AT101 strategy, since AT101 is an inhibitor of antiapoptotic Bcl-2 family proteins and induces cell death in tumor cells including GBMs [26–29]. We showed that cell-type specific responses to TMZ-mediated and also combined TMZ/AT101-induced cytotoxicity occur in different GBM cultures, reflected by individual inductions of dormancy and stemness-associated markers. These augmenting effects of TMZ and AT101 are in accordance with previous findings showing that AT101 could induce caspase-independent, autophagic cell death in malignant glioma cells *in vitro* [28], and that AT101 could inhibit GBM cell proliferation and tumor angiogenesis while enhancing apoptosis in a subcutaneous GBM xenograft mouse model *in vivo* [26]. Now, we complemented these results showing an influence of AT101 on TMZ-induced dormant glioma cells in a cell-type specific extent. While combined TMZ and AT101 treatment usually yielded high cytotoxic effects in GBM cells which responded to small extents to TMZ, additional AT101 treatment mostly induced only slight synergistic cytotoxic effects in GBMs cells responding well to TMZ treatment. Interestingly, AT101 inhibits the hedgehog (Hh) signaling pathway activity, an evolutionarily conserved signaling axis essential for e.g. stem cell regulation, resulting in inhibition of Hh-driven medulloblastoma growth *in vitro* and *in vivo* [29]. In addition, AT101 inhibits colon cancer cell growth by targeting MSI1 resulting in reduced Notch/Wnt signaling and cancer growth [30]. Thus, since we were able to show that TMZ-induced dormant glioma cells also own stem cell characteristics and are less TMZ-sensitive in sequel when stimulated with an additional TMZ-treatment alone, the cytotoxic effects of a combined TMZ/AT101 application may also be attributed to the influence of AT101 on stem cell properties of dormant glioma cells.

Summarized, a close connection between the cellular dormancy of malignancies and the cancer stem cell theory is most likely, and combined treatment strategies seem to be favorable to target tumor cell plasticity in a better way. Overall, we postulate that a better understanding of the dormant state of tumor cells is essential to further improve efficiency of treatment through the prevention of recurrences and thereby to overcome the limitations of current chemotherapeutics.

## MATERIALS AND METHODS

### Human specimens

In total, 74 glioma samples of different entities were surgically obtained at the Department of Neurosurgery (Kiel, Germany) in accordance with the Helsinki Declaration of 1975 and with approval of the ethics committee of the University of Kiel, Germany after written informed consent of donors (file references: D536/15 and D408/14). Tumors were diagnosed and classified according to WHO criteria by a pathologist. Five tumor samples corresponded to astrocytomas WHO II (A II), five to astrocytomas WHO III (A III), and 64 were classified as GBMs WHO IV. If possible (enough material available), matched probes of individual tumor samples were used for experiments.

### Cultivation of GBM cell lines and human primary GBM cells

The human glioblastoma cell lines A172 (ECACC 880624218), U251MG (ECACC 89081403; formerly known as U373MG), and LN229 (ATCC-CRL-2611) were obtained from the European Collection of Cell Cultures (ECACC, Salisbury, UK) or the American Type Culture Collection (ATCC, Manassas, Virginia, USA) and cultured under non-stem cell conditions as described before [31]. Cultured human primary GBM cells were generated by dissociation of tumor material and cultured under non-stem cell conditions as previously described [31], specifications on patients’ and samples’ data are given in Table 1. Different GBM cells were checked for purity by immunostaining with cell type-specific markers and for the absence of *Mycoplasma* contamination. GBM cell lines identity was proven routinely by Short Tandem Repeat profiling at the Department of Forensic Medicine (Kiel, Germany) using the Powerplex HS Genotyping Kit (Promega, Madison, WC) and the 3500 Genetic Analyser (Thermo Fisher Scientific, Waltham, MA, USA).

**Table 1:** Patients’ and samples’ data

ID		27/07	86/13	116/14	118/14	124/15	141/15
**gender**		f	m	m	m	m	m
**age (at surgery)**		38	52	77	65	57	45
**diagnosis**		GBM WHOIV recurrent	GBM WHOIV recurrent	GBM WHOIV primary	GBM WHOIV recurrent	GBM WHOIV primary	GBM WHOIV primary
molecular subtype associated transcription pattern^*^		classical-like	proneural-like	mesen-chymal-like	proneural-like	mesen-chymal-like	neural-like
**clinical background**	**previous diagnosis**	GBM WHO IV recurrent	GBM WHO IV recurrent		GBM WHO IV primary		
	**relapse (after first surgery)**	4 months	2 months		22 months		
	**therapy**	Gliadel-wafer	STUPP		STUPP		
**follow-up**	**therapy**	Gliadel-wafer	Anti-epileptics	STUPP	alternative practitioner	STUPP	STUPP
	**relapse (after last surgery)**	4 months			3 months		22 months
	**last visit (after last surgery)**	4 months	8 months	1 months	3 months	4 days	22 months

^*^Based on determination of PDGFRA, NF1, EGFR and CDKN2A.

### Stimulation of GBM cell lines and human primary GBM cells

1.0 - 5.0 × 10^5^ A172, U251MG, LN229 cells and human primary non-stem GBM cells, respectively, were stimulated with 500 μM Temozolomide [TMZ; Sigma-Aldrich, St. Louis, MO, USA; dissolved in dimethyl sulfoxide (DMSO)] in Dulbecco's modified Eagle's medium (DMEM) supplemented with 10% fetal bovine serum (FBS) for 10-12 days. Controls were stimulated with equal volume [0.2% (v/v)] of DMSO. To prove induction of cellular dormancy, cells were stained with Vybrant® DiO Cell-Labeling Solution (Thermo Fisher Scientific) according to manufacturer's instruction, and dye retention was monitored at day 10 by transmitted-light and fluorescence microscopy with equal exposure times (Zeiss, Oberkochen, Germany) and phospho-p38 / phospho-p42/44 ratios were analysed by Western Blot (see below).

TMZ-sensitivity in sequel of 10-12 days TMZ-pretreated GBM cell was determined by stimulation of 1.0 - 5.0 × 10^5^ LN229 cells with an additional TMZ-treatment (500 μM TMZ, for another 10 days) in comparison to non-pretreated LN229 cells in DMEM supplemented with 10% FBS. Amounts of dead cells were determined using cytotoxicity assays (see below) and calculated as percentage in relation to whole cell numbers (n=3 independent experiments).

For combined chemotherapeutic treatment we used AT101, an inhibitor of antiapoptotic Bcl-2 family proteins, which induces cell death in tumor cells including GBM [26–29]. AT101 (5 μM; TOCRIS, Bristol, UK; R-(-)-enantiomer of Gossypol, dissolved in DMSO) was added (or not in controls) to TMZ stimulated cells at day 6, and stimulation was performed for additional 4-6 days. Morphologies were checked and documented at days 3-6-8-10-12, and RNA was collected at days 3-6-8-10 for qRT-PCR on chosen stem/dormancy markers as described below (at minimum n=4 independent experiments for cell lines). Further, at days 3-6-8-10-12 the relative numbers of dead cells were measured using cytotoxicity assays as described below (at minimum n=3 independent experiments for cell lines). Besides, immunocytochemistry of GBM cell lines was performed at day 10 for chosen stem/dormancy markers as described below (n=2 independent experiments).

### Extreme limiting dilution assay and self-renewal capacity

Self-renewal capacity of 10-12 *versus* 3 days TMZ-pretreated LN229 cells was measured using an extreme limiting dilution analysis (ELDA). Briefly, remaining cells after 10-12 and 3 days of TMZ-treatment were determined, and decreasing numbers (1,600-800-400-200-100-75-50-25-10-5-1 cells per well) of LN229 cells were cultured in neurosphere medium plus 20 ng/ml of basic fibroblast growth factor (bFGF), and 20 ng/ml epidermal growth factors (EGF) as described before [31]. Cultures were maintained until day 10, when the number of spheres per well and wells containing spheres for each cell plating density (number of positive cultures) were recorded and plotted using online ELDA program25 (http://bioinf.wehi.edu.au/software/elda) [32].

### Reverse transcription and quantitative real-time PCR (qRT-PCR)

RNA of tissue and cells was isolated with the TRIzol® Reagent (Invitrogen, Carlsbad, CA, USA) or with the ARCTURUS® PicoPure® RNA Isolation Kit (Applied Biosystems, Waltham, MA, USA) according to the manufacturer's instructions. DNase digestion, cDNA synthesis, and qRT-PCR were performed as described before [31] using TaqMan primer probes (Applied Biosystems): ephrin type-A receptor 5 *(EphA5)* (Hs00300724_m1), glycerinaldehyde 3-phosphate dehydrogenase (*GAPDH*) (Hs99999905_m1), histone cluster 1 H2B family member K *(H2BK)* (Hs00955067_g1), insulin-like growth factor-binding protein 5 (*IGFBP5*) (Hs00181213_m1), Krüppel-like factor 4 *(KLF4)* (HS00358836_m1), musashi (Drosophila) homolog 1 (*MSI1*) (Hs00159291_m1), octamer binding transcription factor 4 *(OCT4)* (Hs00999632_g1), sex determining region Y-box 2 (*SOX2)* (Hs00602736_s1). Cycles of threshold (C_T_) were determined, and ΔC_T_ values of each sample were calculated as CT_gene of interest_ – CT_GAPDH_. Undetectable samples were not included in mean expression calculations. The induction of gene expression upon stimulation is displayed as relative gene expression, n-fold expression changes = 2^ΔCT control - ΔCT stimulus^.

### Immunohistochemistry (IHC) and immunocytochemistry (ICC)

Cryostat sections of GBM tissues and glass cover slips with TMZ and DMSO treated cells were prepared as described before [13, 31]. Primary antibodies were applied overnight at 4°C, secondary antibodies for 1 h at 37°C, nuclei were counterstained, and embedded slides were analysed using a Zeiss fluorescence microscope and a Zeiss camera (Zeiss). Primary antibodies were anti-OCT4 (1:150, ^#^2750, rabbit; Cell Signaling, Danvers, MA, USA), anti-SOX2 (1:200, sc-20088, rabbit; Santa Cruz, Dallas, TX, USA), anti-MSI1 (1:200, MAB2628, mouse; R&D Systems, Minneapolis, MN, USA), anti-KLF4 (1:250 IHC, 1:300 ICC, MA5-15672, mouse; Thermo Fisher Scientific), anti-GFAP (1:500, MAB3402, mouse; Merck Millipore, Billerica, MA, USA), anti-vWF (1:1,000, sc-53465, mouse; Santa Cruz), anti-Ki (Kiel)-67 (Ki67) (1:100, M7240, mouse; Dako, Hamburg, Germany), anti-H2BK (1:400, orb184226, rabbit; Biorbyt, Cambridge, UK), anti-IGFBP5 (1:400 IHC, 1:200 ICC, sc-13093, rabbit; Santa Cruz), anti-EphA5 (1:400 IHC, 1:200 ICC, sc-927, rabbit; Santa Cruz) and anti-CD11b (1:250, sc-1186, mouse, Santa Cruz). If primary antibodies were derived from the same species, unspecific binding was blocked by F(ab) fragments raised against this species (donkey anti-mouse and anti-rabbit F(ab) fragments, 1:1,000, from Jackson ImmunoResearch, West Grove, PA, USA). Primary antibodies were omitted for negative controls. For secondary antibody controls, IgG mouse (MAB002; R&D Systems) or IgG rabbit (AB-105-C; R&D Systems) control antibodies were used instead of primary antibodies at concentrations of the replaced primary antibodies. Donkey anti-mouse or anti-rabbit IgGs labeled with Alexa Fluor 488 or Alexa Fluor 555 (1:1,000; Invitrogen) served as secondary antibodies.

### Western blot

3.0 - 5.0 × 10^5^ A172, U251MG, LN229 and human primary non-stem GBM cells were stimulated with 500 μM TMZ or the equal volume [0.2% (v/v)] of DMSO in DMEM with 10% FBS for 10 days (n=3 independent experiments for glioma cell lines). Cells were harvested, and 3 to 30 μg per sample of protein were used for Western Blotting experiments as described before [31]. Primary antibodies were anti-phospho-p42/44 (1:1,000, ^#^9101, rabbit; Cell Signaling), anti-phospho-p38 (1:100, ^#^4511, rabbit; Cell Signaling), anti-H2BK (1:200, orb184226, rabbit; Biorbyt), anti-IGFBP5 (1:200, sc-13093, rabbit; Santa Cruz), and anti-EphA5 (1:200 IHC, sc-927, rabbit; Santa Cruz), the secondary antibody was donkey anti-rabbit IgG-HRP (1:40,000, sc-2313; Santa Cruz). Equal protein loading was confirmed by stripping and incubating the membranes with anti-extracellular-signal regulated kinase (ERK)-2 (1:200, sc-1647, mouse; Santa Cruz), anti-p38 (1:200; ^#^9212, rabbit; Cell Signaling), anti-glycerinaldehyde 3-phosphate dehydrogenase (GAPDH; 1:250; sc-47724; mouse, Santa Cruz), or anti-heat shock protein 90 (HSP90; 1:1,000; sc-7049; rabbit, Santa Cruz), the secondary antibody was donkey anti-rabbit IgG-HRP or anti-mouse IgG-HRP (1:30,000, sc-2096; Santa Cruz) as described before [31]. Density of kinase signals was measured using PCBAS software, signals of phosphorylated kinases were normalized to unphosphorylated counterparts, and phospho-p38 / phospho-p42/44 ratios were calculated for DMSO and TMZ treated samples.

### Cytotoxicity assay

Cytotoxic effects of TMZ, AT101 and DMSO treatment on A172, U251MG, LN229 cell lines and human primary GBM cells (27/07, 86/13, 116/14, 118/14, 124/15, 141/15) were investigated with the CytoTox-Fluor^TM^ Cytotoxicity Assay (Promega) according to the manufacturer's instruction. Briefly, supernatants of different TMZ-stimulated and control cells were collected at days 3-6-8-10-12, mixed with the bis-AAF-R110 substrate and measured in a fluorescence microplate reader (GENios, TECAN, Zürich, Switzerland). DMSO-treated cells served as negative controls, as positive controls defined numbers of Digitonin-lysed (Merck Millipore) glioma cells were used. Numbers of dead cells were determined according to this internal standard curves and displayed as additive n-fold cytotoxic effects. The combinational index of cytotoxic effects was calculated using the CompuSyn software which allows a computerized simulation of synergism, additive and antagonism in drug combination studies (http://www.combosyn.com/) [33, 34].

### Statistical analysis

For statistical analysis a two-tailed Student's *t*-test was used. Significance levels were p<0.05 (^*^), p<0.01 (^**^) and p<0.001 (^***^).

## SUPPLEMENTARY MATERIALS FIGURES



## Abbreviations

A: astrocytoma
C_T_: cycle of threshold
DMEM: Dulbecco's modified Eagle's medium
DMSO: dimethyl sulfoxide
ELDA: extreme limiting dilution analysis
EphA5: Ephrin type-A receptor 5
FBS: fetal bovine serum
GAPDH: glycerinaldehyde 3-phosphate dehydrogenase
GBM: glioblastoma multiforme
GFAP: glial acidic fibrillary protein
H2BK: histone cluster 1 H2B family member K
HSP90: heat shock protein 90
ICC: immunocytochemistry
IGFBP5: insulin-like growth factor-binding protein 5
IHC: immunohistochemistry
Ki67: antigen Ki (Kiel)-67
KLF4: Krüppel-like factor 4
MSI: musashi (Drosophila) homolog 1
OCT4: Octamer binding transcription factor
qRT-PCR: quantitative reverse transcription polymerase chain reaction
SOX2: sex determining region Y-box 2
TMZ: Temozolomide
vWF: von Willebrand factor protein
WHO: World Health Organization.
