# Supplementary material for: Dormant glioblastoma cells acquire stem cell characteristics and are differentially affected by Temozolomide and AT101 treatment
Source: Oncotarget. 2017 Nov 18;8(64):108064–78. doi: 10.18632/oncotarget.22514 (PMC5746126; doi:10.18632/oncotarget.22514)
Supplement: Supplementary file 1 [file oncotarget-08-108064-s001.pdf]

Dormant glioblastoma cells acquire stem cell characteristics and are differentially affected by Temozolomide and AT101 treatment

SUPPLEMENTARY MATERIALS

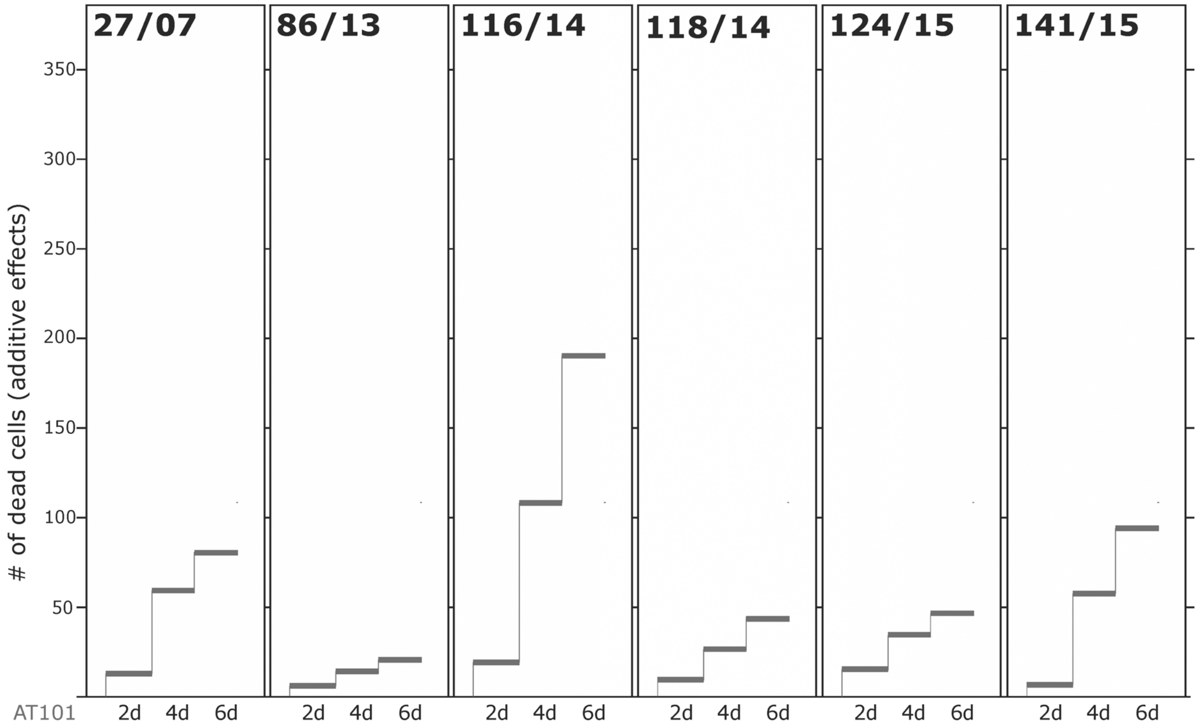

Supplementary Figure 1: Determination of AT101-induced cytotoxicity in human GBM primary cultures. Non-stem primary cultures were stimulated with 5 µM AT101 or 0.005% DMSO (control) for up to 6 days. Numbers of dead cells were determined at days 2-4-6 and documented as n-fold cytotoxic effects.

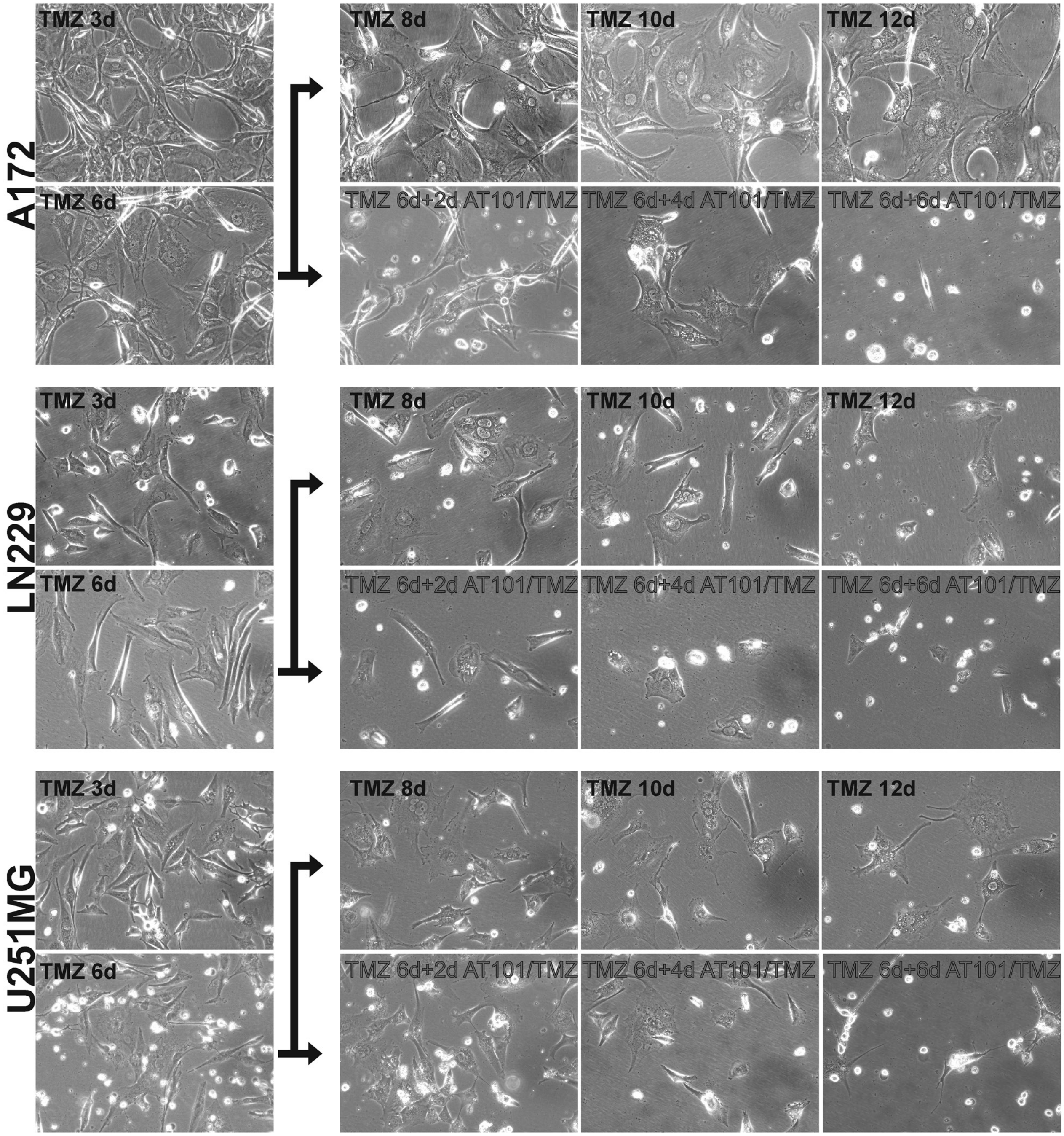

Supplementary Figure 2: Schedule of TMZ and TMZ/AT101-combined treatment of GBM cells. Non-stem GBM cells were stimulated with 500 µM TMZ, 5 µM AT101 or 0.2% DMSO (control) for up to 12 days. 5 µM AT101 was added to TMZ stimulated cells at day 6, and stimulation was performed for additional 4 to 6 days. Morphologies and amounts of DMSO, TMZ and TMZ/AT-101-treated GBM cells were documented at days 3-6-8-10-12.

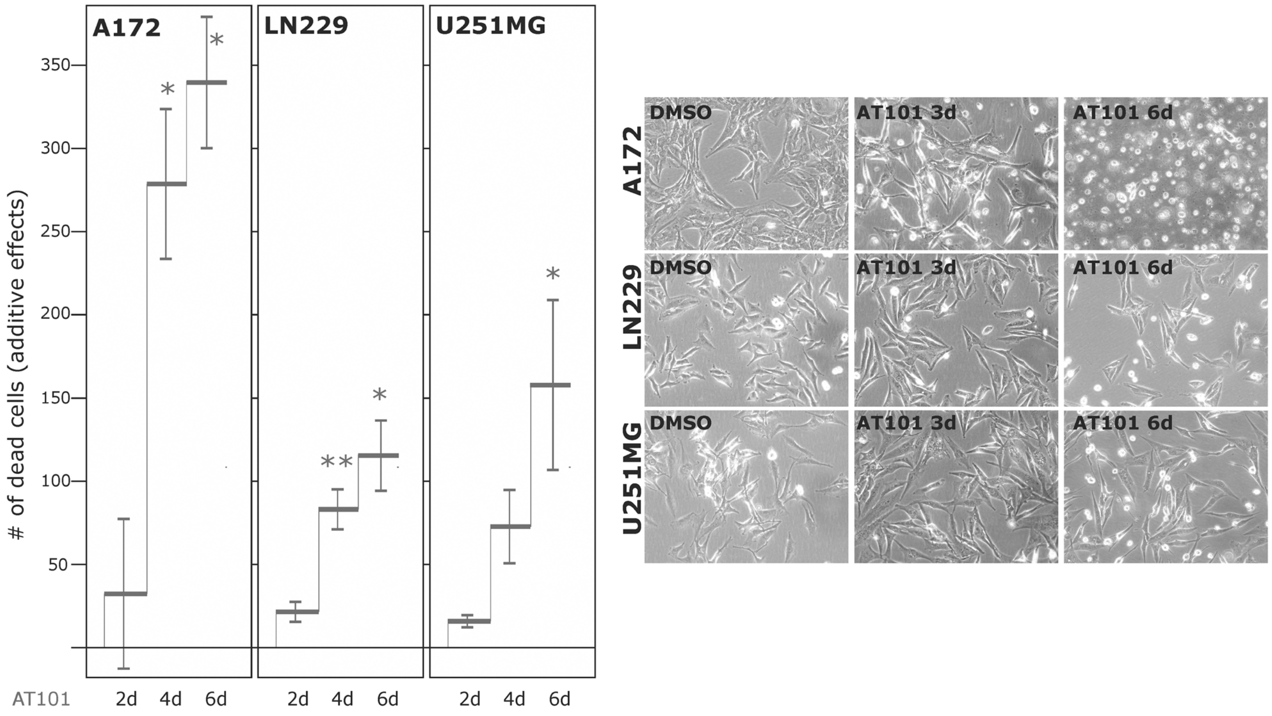

Supplementary Figure 3: Determination of AT101-induced cytotoxicity in glioma cell lines. Non-stem GBM cell lines were stimulated with 5 µM AT101 or 0.005% DMSO (control) for up to 6 days. Numbers of dead cells were determined at days 2-4-6 and documented as n-fold cytotoxic effects, and morphology of treated GBM cells was documented at days 3 and 6 (*p<0.05, **p<0.01).
